# Automated migration analysis based on cell texture: method & reliability

**DOI:** 10.1186/1471-2121-6-9

**Published:** 2005-03-03

**Authors:** Jianfeng Qin, Thomas W Chittenden, Ling Gao, Justin D Pearlman

**Affiliations:** 1 Thayer School of Engineering, Dartmouth College, Hanover, NH, 03755, USA; 2 Dartmouth Advanced Imaging Center, Dartmouth-Hitchcock Medical Center, One Medical Drive, Lebanon, NH, 03756, USA; 3 Angiogenesis Research Center, Dartmouth-Hitchcock Medical Center, One Medical Drive, Lebanon, NH, 03756, USA; 4 Dartmouth Medical School, Hanover, NH, 03755, USA

## Abstract

**Background:**

In this paper, we present and validate a way to measure automatically the extent of cell migration based on automated examination of a series of digital photographs. It was designed specifically to identify the impact of Second Hand Smoke (SHS) on endothelial cell migration but has broader applications. The analysis has two stages: (1) preprocessing of image texture, and (2) migration analysis.

**Results:**

The output is a graphic overlay that indicates the front lines of cell migration superimposed on each original image, with automated reporting of the distance traversed vs. time. Expert preference compares to manual placement of leading edge shows complete equivalence of automated vs. manual leading edge definition for cell migration measurement.

**Conclusion:**

Our method is indistinguishable from careful manual determinations of cell front lines, with the advantages of full automation, objectivity, and speed.

## Background

The images we deal with are images of endothelial cells. Endothelial cells migrate to sites of injury in the body. They are involved in forming new blood vessels to help repair damaged areas [1,2].

In particular, we are interested in the effects of SHS on endothelial migration. By comparing automated migration analysis with varied exposure to SHS, for cells with and without specific genes, we can examine why exposure to SHS impairs endothelial cell migration and explore possible cures [3,4].

**Figure 1:**
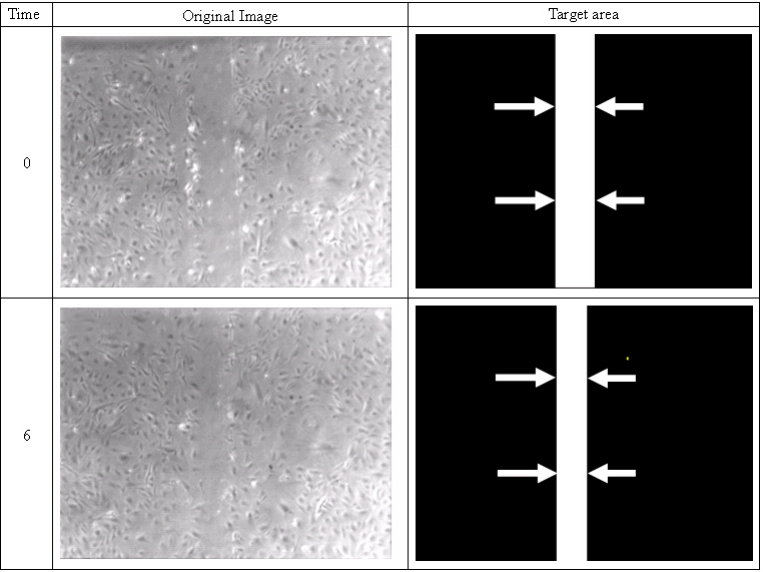
Images of a plate of endothelial cells growing in agar. Cells appear as dark spots and contrast is limited because introduction of stains to increase contrast could affect cell function. An early time image (top row) shows a wide trough where a lane of cells was removed by the experimenter. A later time points (bottom row) show the effect of progressive narrowing of this lane as the cells migrate to fill in the gap. Our goal is to measure the width of the "clear lane" which corresponds to the amount of cell migration in the time interval.

Cell migration is a basic biologic function that can be modified by changes in genetic code and in response to chemical and other stimuli. Upon 24 hours serum starvation, the cells were artificially wounded using P20 pipette tip across the plate, then cultured respectively in regular DMEM or DMEM containing Second Hand Smoke (SHS) (unpublished). The subsequent gaps were imaged at 0 and 6 hours post SHS exposure as previously described to determine the rate of migration of the front lines to close in the gap [5-8,23]. Figure 1 gives an example about the cell migration.

The automated borders are compared blindly by a team of domain experts to manual borders created by a technician to assess accuracy. Results are also evaluated blind to biologic significance to determine concordance and power to demonstrated biological effects.

Biologists deal with this by making multiple manual measurements, to report an average. Observers have difficulty deciding where and how many times to measure the width. Besides, there are many pairs of images to be processed. Therefore, automatic measurement is desired.

## Implementation

### Generating texture

The primary difference between cell-populated areas and the clear lane is texture. The cell-populated areas are speckled with cells, the clear lane is not.

In order to capture the cellularity characteristic of the source images, we sought to compute a texture index that would emphasize the cellular attribute of the region of interest and also minimize the influence of non-cellular signal variations [11-14]. Because the image may have non-uniform background where the "clear lane" can be 'darker' than the 'cells' at other locations, the texture index should be generated from the relative gray value difference. Furthermore, we know the orientation of the experimentally produced clear lane, which we take to be vertical. Then our algorithm generates the texture in this way:

For each point in the original image

Search for darker point in this line vertically

If find

Set the distance between start and darker point as the gray value of the corresponding point in the texture map

Search for continual darker points and set the distance as the value of them Scale the value to 0–255:



where pv is the new pixel intensity value, cv is the distance value of the corresponding point and max and min are the minimum and maximum distance value.

**Figure 2:**
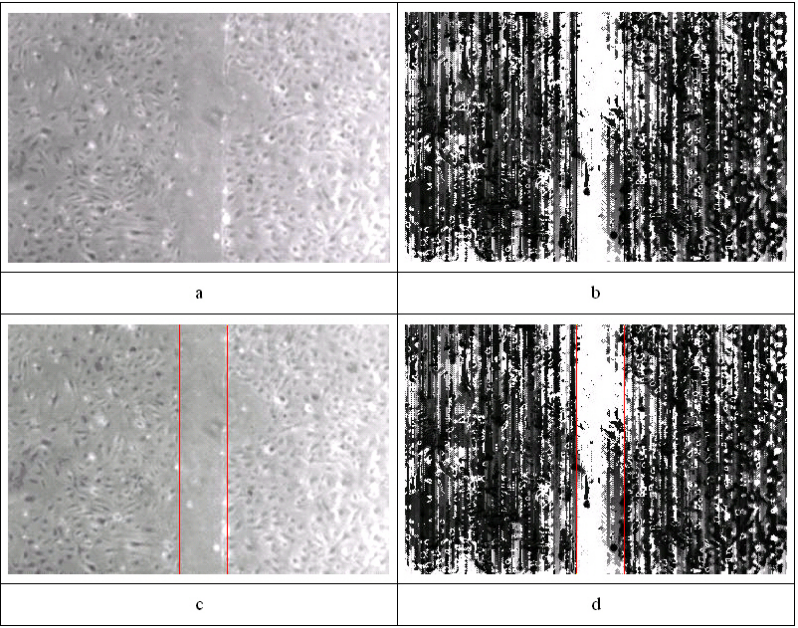
An example image is shown in panel (a), followed by the derived texture index (b) and the resultant graphic overlay (c & d). Note that the region between the vertical lines of 2D is relatively devoid of cells, and each line represents the front of cell migration, as desired.

Panel b of figure 2 shows the texture of Panel a of figure 2 using this algorithm.

### Migration analysis

Based on the texture map, the region we are interested in appears as a white vertical band. Thus the second stage of analysis must determine the position and width of this lane. As the information in each vertical column is equivalent to repeated measures, we can combine the data to marginal projection. From the histogram of this we can compute a classifier for lane vs. cells and determine the half-height width. The locations are then mapped to a graphic overlay on the original image to demarcate the front lines of cell migration. The change in distance between the front lines reports the amount of (bi-front) cell migration.

**Figure 3:**
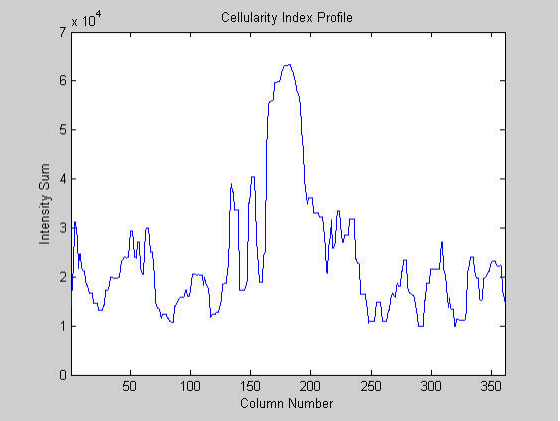
The marginal cellularity index profile.

1. Project the texture values to a marginal profile "cellularity index profile" (Figure 3) which is an array of P --- P [1..*n*] where n is the width of the image. The value of each P [*i*] is the intensity sum of all the pixels in i column.

2. Compute the discriminant classifier (DC) which is average value of P [*i*].

3. Locate the leading and trailing edges based on classifier crossing. If P [*i*] < DC and P [*i*+1] > P [*i*], i is the leading edge. Conversely, if P [*i*] > DC and P [*i*+1] < DC, i is the trailing edge. Then a few pairs of leading and trailing edges could be obtained. The target pair is identified based on the width, P [*i*] values between leading and trailing edges and "clear lane" location in the time neighboring image.

4. Record locations and generate graphic overlay for original image.

### Validation

Since the manual assessment is the research gold standard for image processing [15-18], a technologist specially trained to identify the leading and trailing edges of cell migration was provided a computer tool to mark those edges manually in a manner compatible with the graphics overlay engine. These are called "manual edges." The manual edges and the automated edges were then presented to a team of domain experts in random order pairs (one of each on corresponding image) for preference scoring. The scores ranged from 1–5, where 1 is strong preference for first overlay, 2 mild preference for first overlay, 3 equivalency, 4 mild preference for second overlay, and 5 strong preference for second overlay. Results were analyzed by Kappa statistic as a measure of agreement.

## Results

**Figure 4:**
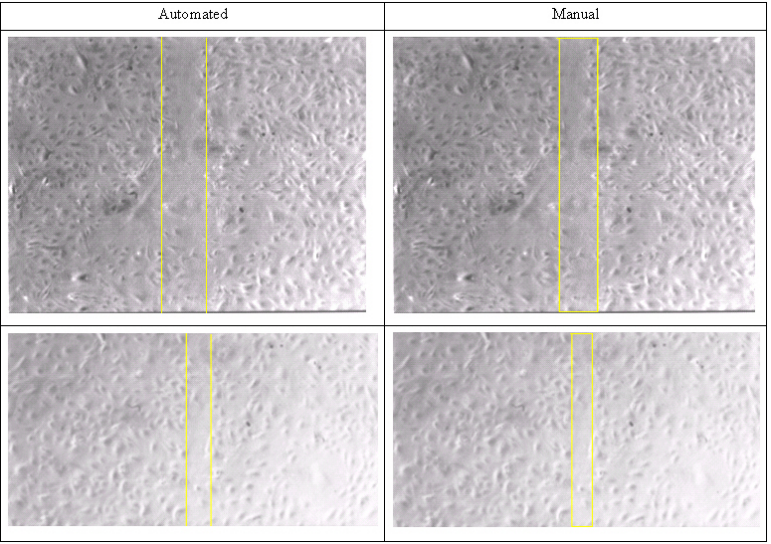
Examination of width differences between automated and manual identified two outliers with relatively large differences. These are shown as two pairs. Note the uneven heterogeneous cell distribution in these cases, a result that is technical suboptimal and not desired, likely from an error in the excoriation (creation of clear channel). These are a poor sample pairs for technical reasons, and in retrospect domain expert still had no significant difference in preference of manual result over the automatic result.

After analysis, results like the ones shown in the Figure 2 are obtained. Panel (a) shows a photograph of the cell cultures, while the remaining panels show various aspects of the analysis. Figure 4 shows the two worst cases of disagreement between automated and manual methods.

The results of domain expert preference by quality for automated vs. manual assignation of migration front lines, evaluated blinded to method, randomized, and subsequently decoded. Overall, there is complete equivalence of automated vs. manual with respect to expert preference for quality. The values ranged 2–4. In no cases was manual strongly preferred over automatic. Preference testing of analysis methods showed near equivalence, favoring preference for the automated borders (3.02 ± 0.11). Agreement between observers in preference was examined for two domain experts, revealing good agreement (Kappa = 0.59, p < 0.003). Agreement in preferences by a technologist without domain expertise was lower (Kappa = 0.23, 0.25, p > 0.10) but supported the same conclusion: the automated analysis is at least as good as manual selection by domain experts.

**Figure 5:**
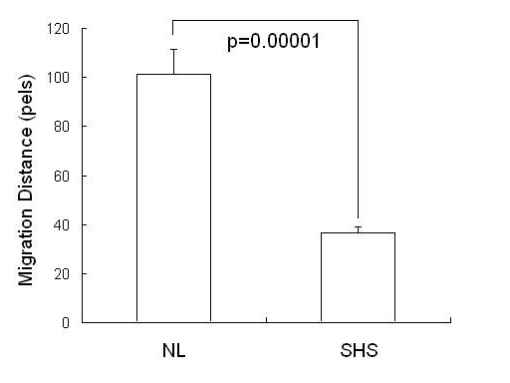
The migration distance of control group (NL) is 101.30 ± 10.32 and distance of SHS group 36.25 ± 2.71. Pair t test shows two groups are obviously different with p value = 2.24E-06.

Application of this method to determine the effect of SHS on endothelial cell migration demonstrates that SHS can reduce the cell migration rate, which is statistically significant (Figure 5).

## Discussion

Our migration analysis is based on the texture index of the images. This index should reflect the attribute of the images. Since no global thresholding technique could be used in our images, the segmentation of regions and boundaries (edges) have to consider the local property [9]. Because the target boundaries always show as a vertical band, the line-based segmentation appears to be the most suitable approach for our task. Further analysis of regions and edges is based on a uniform data structure reflecting the texture character in each column.

Our results show a robust automatic method with no detected errors. This study is a pilot study demonstrating feasibility and biologic significance in real application. Further collective experience in multi-center applications are needed to establish the full utility of the method.

In addition, the program runs on the software platform, ImageJ [10] and the speed is fast. A normal process time for one study of images is less than 3 minutes. Results such as width and percentage can be shown as a table. It offers a convenient way for researcher to process their image data using excel.

## Conclusion

We describe a novel method of cell migration analysis based on texture pre-processing and discriminant analysis. Domain expert preference testing demonstrates that this automated method compares favorably to the much more painstaking manual method.

The further study is to apply this to evaluation of the impact of SHS on endothelial cell migration. For that purpose, we have constructed a SHS capture system in which we bubble the SHS through tissue culture medium to assess its impact on cell migration. Our results indicate that this analysis system is very sensitive to biological effects, documenting that SHS impairs cell migration [19-22].

## Availability and requirements

Project name: Cell migration measurement project

Project home page: 

Operating system(s): Platform independent

Programming language: Java

Other requirements: Java 1.3.1 or higher, ImageJ

License: Null

Any restrictions to use by non-academics: Licence needed

## Authors' contributions

JDP proposed and designed the method to evaluate cell migration objectively and automatically. JQ implemented the method as a plugin of ImageJ and validated the method. TWC performed the cell experiments and captured the images. LG performed manual edge definition and subsequently performed statistic analysis of expert preferences. All authors read and approved the final manuscript.
